# Impact of Helminth Infections on Female Reproductive Health and Associated Diseases

**DOI:** 10.3389/fimmu.2020.577516

**Published:** 2020-11-23

**Authors:** Alisha Chetty, Millicent A. Omondi, Claire Butters, Katherine Ann Smith, Gnatoulma Katawa, Manuel Ritter, Laura Layland, William Horsnell

**Affiliations:** ^1^ Institute of Infectious Disease and Molecular Medicine and Division of Immunology, University of Cape Town, Cape Town, South Africa; ^2^ School of Biosciences, Cardiff University, Cardiff, United Kingdom; ^3^ Ecole Supérieure des Techniques Biologiques et Alimentaires, Université de Lomé, Lomé, Togo; ^4^ Institute for Medical Microbiology, Immunology and Parasitology (IMMIP), University Hospital Bonn (UKB), Bonn, Germany; ^5^ Institute of Immunology and Immunotherapy, University of Birmingham, Birmingham, United Kingdom

**Keywords:** Helminths, female reproductive tract, sexually transmitted infections, fertility, Systemic immunity

## Abstract

A growing body of knowledge exists on the influence of helminth infections on allergies and unrelated infections in the lung and gastrointestinal (GI) mucosa. However, the bystander effects of helminth infections on the female genital mucosa and reproductive health is understudied but important considering the high prevalence of helminth exposure and sexually transmitted infections in low- and middle-income countries (LMICs). In this review, we explore current knowledge about the direct and systemic effects of helminth infections on unrelated diseases. We summarize host disease-controlling immunity of important sexually transmitted infections and introduce the limited knowledge of how helminths infections directly cause pathology to female reproductive tract (FRT), alter susceptibility to sexually transmitted infections and reproduction. We also review work by others on type 2 immunity in the FRT and hypothesize how these insights may guide future work to help understand how helminths alter FRT health.

## Burden of Disease

Helminth infections are widespread and are characterized by sophisticated host immune modulation and evasion. Helminth infections are a global health concern, with more than 1.7 billion affected worldwide, particularly in tropical and subtropical regions (1). A feature of helminth infections are the parasites’ ability to alter immunity and susceptibility to unrelated diseases (2–7). Of particular interest is the potential impact of helminth immune-regulation on susceptibility to sexually transmitted infections (STIs), given their high incidence in developing regions and detrimental impact on public health (8). For example, Ivan et al. (9) studied a cohort of 328 Rwandan pregnant women on anti-retroviral therapy, 38% of whom were stool positive for helminth infections (9). Mkhize-Kwitshana et al. (10) reported 66% of HIV+ study participants from an helminth endemic region of South Africa, were helminth egg positive and/or helminth-specific IgE seropositive (10). Likewise, Abossie and Petros (11) reported 68% of study participants in Ethiopia were co-infected with helminths and HIV, 35% were women (11). In this review we address how the geographical overlap between helminth exposure and STIs can result in parasite-induced changes to female reproductive health (12–14).

## Helminth Immunity

Host immunity to helminths has been studied in depth using mouse models reflective of human infection and immunity (15–18). Typically, helminths induce a type 2-skewed immune response, associated with the production of the canonical cytokines interleukin (IL)-4, IL-5, and IL-13 (19–26). These cytokines amplify alternatively activated macrophages (AAMs; M2) (27–29), eosinophilia (30–32), smooth muscle contraction and goblet cell hyperplasia; cellular and physiological responses that underlie the ‘weep and sweep’ worm expulsion from the intestine (21, 23, 24, 26, 33, 34). Consistent with *in vivo* studies, epidemiological studies also report type 2-biased immune responses in humans infected with roundworm *Ascaris lumbricoides* (35–37), whipworm *Trichuris trichiura* (36–38), and hookworm *Necator americanus* (39). Furthermore, experimental infections of participants with hookworm has been shown to result in strong mucosal and systemic type 2 cytokine responses (40). Helminth infections also elicit regulatory immune responses, characterized by transforming growth factor-β (TGF-β), IL-10 and expansion of FoxP3-expressing regulatory T cells, involved in immune polarization and controlling inflammation (2, 41–48).

Antagonism between type 1 and type 2 immunity is central to our understanding of the T helper (Th) 1 cells (Th1)- T helper 2 cells (Th2) immune paradigm: Mosmann et al., first described Th1 and Th2 CD4^+^ T cell differentiation and cytokine responses (49, 50), and Fernandez-Botran et al. (51) first demonstrated Th subtype regulation of each other (51). Furthermore, Reese et al. (52) demonstrated that IL-4 and STAT6 signaling can competitively inhibit interferon (IFN)-γ production (52). This paradigm has been expanded beyond T cell responses, as what is known as type 1 and type 2 immunity and regulation. For example, AAMs are a key feature of helminth infection induced by IL-4, -13 and -10. AAMs synthesize high levels of the enzyme arginase-1, which inhibits nitric oxide (NO) production (53). In addition, AAMs downregulate inflammatory Th1 immune responses mediated by TGF-β (54), which induce the development of regulatory T cells (41). Considering the opposing responses of type 1 and type 2 immunity, it is hypothesized that canonical type 2 immunity induced by helminths, can influence Th1- and Th17-mediated immune protection against STIs in the female reproductive tract (FRT).

## Helminth-Induced Immune Modulation

Co-evolution of parasitic worms with the host is thought to have resulted in their ability to evade host’s immunity through highly sophisticated responses. Helminths actively promote the expansion of regulatory T cell populations, promoting helminth persistence as well as host survival following infection (41, 44, 45, 55, 56). This can be achieved by the helminths release of excretory/secretory products, which effectively target and inhibit specific components of anti-parasite immune mechanisms or induce favorable immune regulation (43). For example, *Heligmosomoides polygyrus* excretory/secretory products (HES) contain a TGF-β mimic, the importance of this is supported by blockade of HES TGF-β mimic *in vivo* resulting in parasite expulsion in susceptible C57BL/6 mice (41). Bancroft et al. (57) recently identified the immunomodulatory molecule p43, a major secreted protein by murine whipworm *T. muris*, which binds to and inhibits IL-13 activity (57). Helminth-induced immune modulation benefits parasite survival by supporting asymptomatic or chronic infections. This has been demonstrated by individuals with asymptomatic lymphatic filariasis who display regulatory T and B cell responses (58), as well as skewed Th2 and regulatory T cell cytokine profiles i.e. favorable IL-4 and TGF-β, over IFN-γ and IL-17 production (46, 59–61). Alternatively, symptomatic patients had dominant pro-inflammatory responses, i.e. Th1, Th17 inflammatory responses and uncontrolled Th2 responses, resulting in immune-mediated damage of colonized tissue leading to severe symptoms like dermatitis in hyperreactive onchocerciasis or elephantiasis in lymphatic filariasis (62, 63).

Importantly, helminth-induced immune modulation has bystander effects on unrelated conditions such as allergies, autoimmune and inflammatory disorders, and unrelated infections. McSorley et al. (2) reported the suppression of type 2 allergic lung inflammation from treatment with HES (2), associated with TGF-β-like activity (41). Furthermore, Johnston et al. (42) demonstrated the suppression of skin allograft rejection by treatment with a TGF-β mimic isolated from HES (42). In support, Li et al. (64) demonstrated suppression of allograft rejection with *H. polygyrus*-induced Th2 and regulatory T cell bystander immunity (64). Recombinant hookworm anti-inflammatory proteins have been shown to reduce inflammation during experimental colitis (65) and asthma (66), associated with the induction of regulatory T cells. Layland et al. (67) demonstrated the suppression of allergic airway inflammation mediated by *S. mansoni*-induced regulatory T cells *in vivo* (67). Furthermore, Straubinger et al. (68) showed reduced susceptibility to ovalbumin (OVA)-induced allergic airway inflammation in mice born to mothers infected with *S. mansoni* during pregnancy. Osbourn et al. (69) described the ability of *H. polygyrus* Alarmin Release Inhibitor (HpARI) secreted protein to bind to and suppress IL-33 activity, reducing ILC2 and eosinophil responses, and promoting parasite survival (69). Interestingly, Zaiss et al. (70) demonstrated that infection with GI *H. polygyrus* resulted in changes to host intestinal microbiota and increased microbial-derived short chain fatty acids, which contributed to helminth-induced suppression of allergic lung inflammation (70). Conversely, Pinelli et al. (71) reported exacerbated ova-induced allergic airway inflammation in mice infected with *Toxocara canis* (71). In humans, Jõgi et al. (5) reported increased risk of allergy manifestations in Norwegian children with anti-*T.* *canis* IgG4 seropositivity (5).

In addition to modulation of allergies and autoimmunity, Darby et al. (72) recently demonstrated how pre-conception maternal helminth exposure influences offspring immunity to helminth infection. Prior murine hookworm, *Nippostrongylus brasiliensis* infection imprinted Th2 immunity in female mice, which was transferred *via* breast milk and conferred protection against the parasite in their offspring. Protection was associated with maternally-derived Th2 primed CD4^+^ T cells (72). Helminth-induced bystander immunity has also been implicated in altered vaccine responses (73–77) and immunity to unrelated infections. This highlights the potential significance of a transgenerational axis of influence on immunity by helminth infections.

Helminth-induced bystander immunity has also been implicated in altered vaccine responses and immunity to unrelated infections (73–77). For example, mouse infection with *T. spiralis* and *H. polygyrus* can impair immunity to murine norovirus (MNV) in the co-colonized intestine, mediated through impaired type 1 responses by type 2 activation of macrophages (78). Changes to lymphoid lineage function are demonstrated by Rolot et al. (7), who show helminth-mediated expansion of virtual memory CD8^+^ T cells which enhance control of subsequent murine γ-HV respiratory infection (7). McFarlane et al. (79) showed that infection with murine nematode *H. polygyrus*, altered gut microbiota, which systemically increased proinflammatory type I IFN, and protected against subsequent respiratory viral infection (79). Additionally, *in vivo* infection with *T. spiralis* reduced pathological inflammation of the airways following influenza A virus infection (80). Helminth infection also impacts on control of bacterial infections. *N. brasiliensis* infections have been shown to impair natural and vaccine elicited T cell and B cell responses against *Salmonella typhimurium* infection *in vivo* (4). Protection against bacterial infections has also been reported; with reduced pulmonary mycobacterial burdens during concurrent nematode infection in mice, that required helminth-modified alveolar macrophage responses (3). Human studies have also identified helminth-associated changes to myeloid responses that relate to protection against MTb. For example, a negative association between hookworm infection and latent Mtb infection in Nepalese immigrants to the UK, was associated with elevated eosinophil numbers (6). Coincidence of filarial infection has also been associated with moderate protective immunity during latent Mtb infection (62, 81) and in a recent study, *S. stercoralis* infection in latent tuberculosis patients, was associated with down-regulated chemokine responses (82). Associations between soil-transmitted helminth (STH) infection and higher risk of concurrent bacterial and protozoal infections, and lower risk of concurrent viral infections in children and adults have also been reported (83). Recent studies have also demonstrated that prior nematode infection can confer resistance to subsequent infection by a different nematode species (84, 85). Together this existing body of work shows that helminths infections can have diverse influences on unrelated disease at sites distal to the anatomical location of the helminth in the host.

## Helminths, Female Reproductive Tract, and Susceptibility to STIs

Immune imprinting on helminth infected hosts is therefore a feature of tissues not colonized by the parasite (86), including the FRT (87). The impact of helminth infection on immunity in the FRT and subsequent immune responses to sexually transmitted infections is not well studied, but it is apparent that significant effects on disease control in the FRT are likely.

### Immune Control of STIs

The vaginal mucosa, the entry point for most STIs, is a unique and dynamic mucosal site under the cyclic influence of female sex hormones, and is made up of stratified squamous epithelial, lined by mucous, commensal bacteria and other anti-microbial defenses (88–91). In addition, the vaginal submucosa is surveyed by resident immune cells such as dendritic cells (DCs), which mount the response against invading pathogens (92–95). Host immune control of STIs is strongly correlated with the pattern of cytokine production in the host. Differential activation of Th1 cells, producing IL-2 and IFN-γ, mediate cellular immune responses, whereas Th2-like cells producing IL-4, IL-5, and IL-13, facilitate humoral immunity (96). Persistence of STIs can also be influenced by the production of IL-10 (97) and activation of regulatory T cells (98). While many STIs are initially asymptomatic, lack of treatment can result in an increased risk of acquiring another STI, infertility, organ damage, cancer, or death.

**Figure 1:**
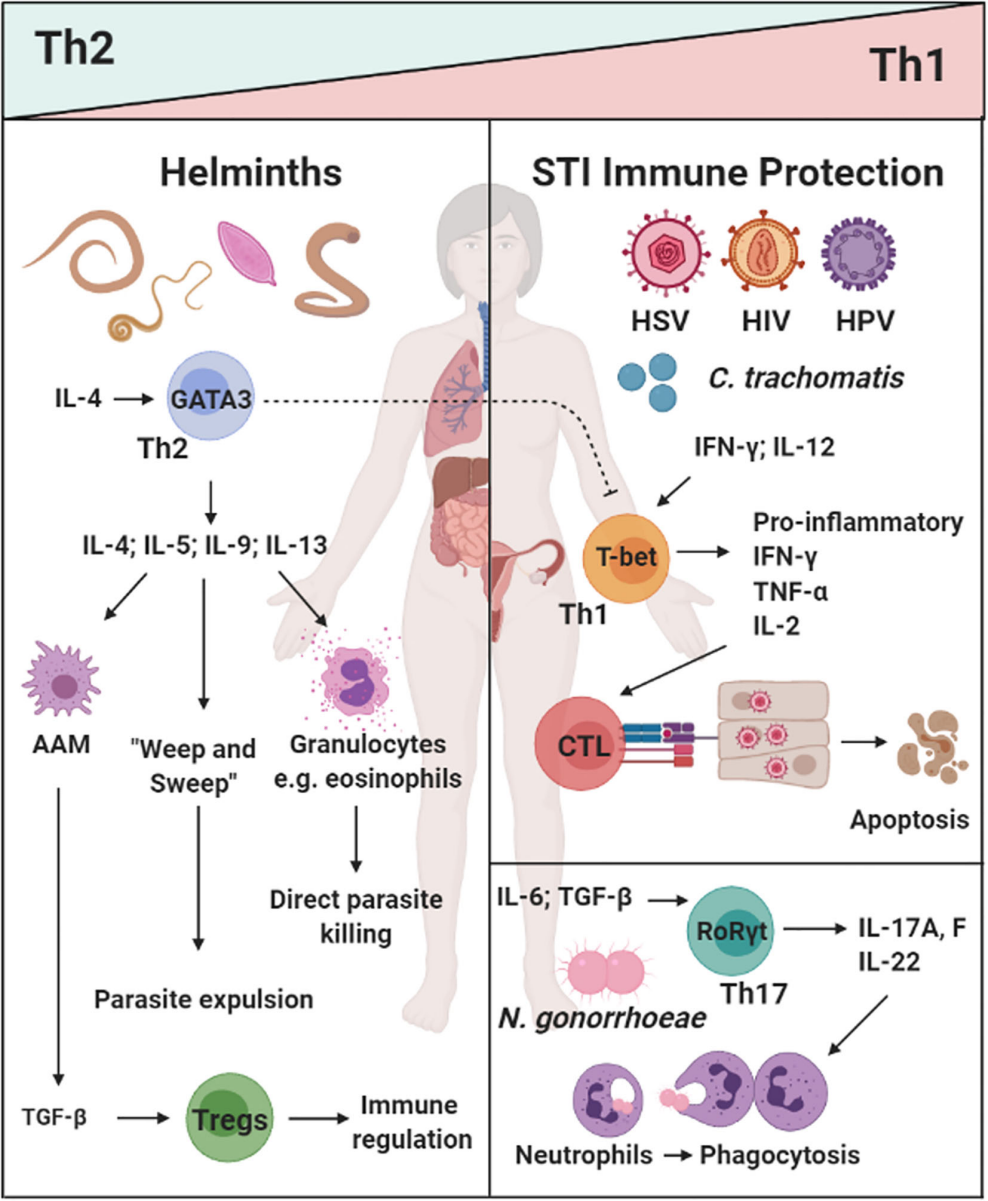
The dichotomy of helminth-induced Th2/type 2 and regulatory immunity, and protective responses against sexually transmitted infections (STIs) in the female reproductive tract (FRT): Helminth infections (e.g. *A lumbricodes, T. trichiura, Schistome eggs*) commonly induce a potent Th2/type 2 immune response characterized by type 2 cytokines IL-4, IL-9, and IL-13, which induce a potent type 2 effector cells and functions (e.g. eosinophils, alternatively activated macrophages(AAMs), “weep and sweep” responses) (20, 21, 35, 36, 38–40). Prevalent viral [Herpes Simplex Virus type II (HSV-2), Human Immunodeficiency Virus (HIV), and Human Papillomavirus (HPV)] and bacterial (*C. tranchomatis and N. gonorrhoeae*) vaginal infection are a serious health concern for women in low- and middle-income countries (LMICs). Protective immunity against these pathogens can be classified a Th1/type 1 and Th17 responses i.e. cytotoxic killing of infected cells or phagocytosis of extracellular pathogens (101–107–109). How helminth exposure and immune modulation may influence susceptibility and control of STIs, is not fully understood. Created with BioRender.com.

The most common sexually transmitted viral infections (STVIs) of the FRT are Herpes Simplex Virus type II (HSV-2), Human Papillomavirus (HPV) and Human Immunodeficiency Virus (HIV). Control of STVIs is typically associated with type 1 immune responses (99, 100). With the exception of HIV, killing of virally infected cells requires Th1 polarization of CD4^+^ T cells (101), production of type 1 cytokines such as IFN-gamma (IFN-γ) (102, 103) and cytotoxic T cell responses (104–106) (**Figure 1**). Th1 immunity is also critical for early control of HIV, however, this response is insufficient to resolve infection (110), due to the virus’ ability to rapidly mutate and evade CD8^+^ T cell responses (107). Pre-existing inflammation and increased presence of CD4^+^ target T cells in the FRT are major risk factors for increased susceptibility to HIV infection (111). Elimination of CD4^+^ T cells by HIV is a hallmark of acquired immune deficiency syndrome (AIDS), resulting in increased susceptibility to opportunistic infections (112) and viral-associated cancers (113).

Similarly to STVIs, bacterial infections of the FRT require a Th1 and/or Th17 response to clear the infection (114, 115). *Chlamydia trachomatis* is a common bacterial STI worldwide, with women carrying the burden of this disease (116). IFN-γ production by Th1 CD4^+^ T cells have been shown to be important for the resolution of *C. trachomatis* infections (117, 118). Cytotoxic T lymphocyte (CTL) responses are not required for clearance of this infection and instead have been shown to promote tissue pathology in the upper genital tract (108, 109). Another common bacterial STI is *Neisseria gonorroeae*, the causative agent of gonorrhea. In a murine model of infection, Th17 immune responses were shown to be favorable for *N. gonorrhoeae* clearance (114). Considering the established counterbalance between Th2/Treg immunity and Th1/Th17 responses (50, 52, 119, 120), it is important to understand the consequence of helminth-induced immunity on susceptibility to co-endemic STIs (**Figure 1**).

### Genital Schistosomiasis

*Schistosoma haematobium* infections have profound effects on female genital health. *S. haematobium* larvae (cercariae) emerge from aquatic snails and infect the human host through skin penetration. The larvae develop into schistosomula and migrate through the vasculature. Eventually, these mature into adult parasites, pair up and reside for years in the pelvic venous plexus. *S. haematobium* eggs produced here lodge in the urinary bladder wall and FRT, causing urogenital schistosomiasis (121). In chronically infected individuals, vaginal pathology here is acute with reported itching, pain, hematuria and ulceration in *S. haematobium*-infected individuals (122–125). Pathology is driven by eggs traversing host tissue and the formation of calcified granulomas in the female urinary and reproductive tract. The World Health Organization (WHO) International Agency for Research on Cancer (IARC) declared *S. haematobium* a group 1 carcinogen, as the correlation between urogenital schistosomiasis and the occurrence of bladder cancer has been extensively proven (126).

**Figure 2:**
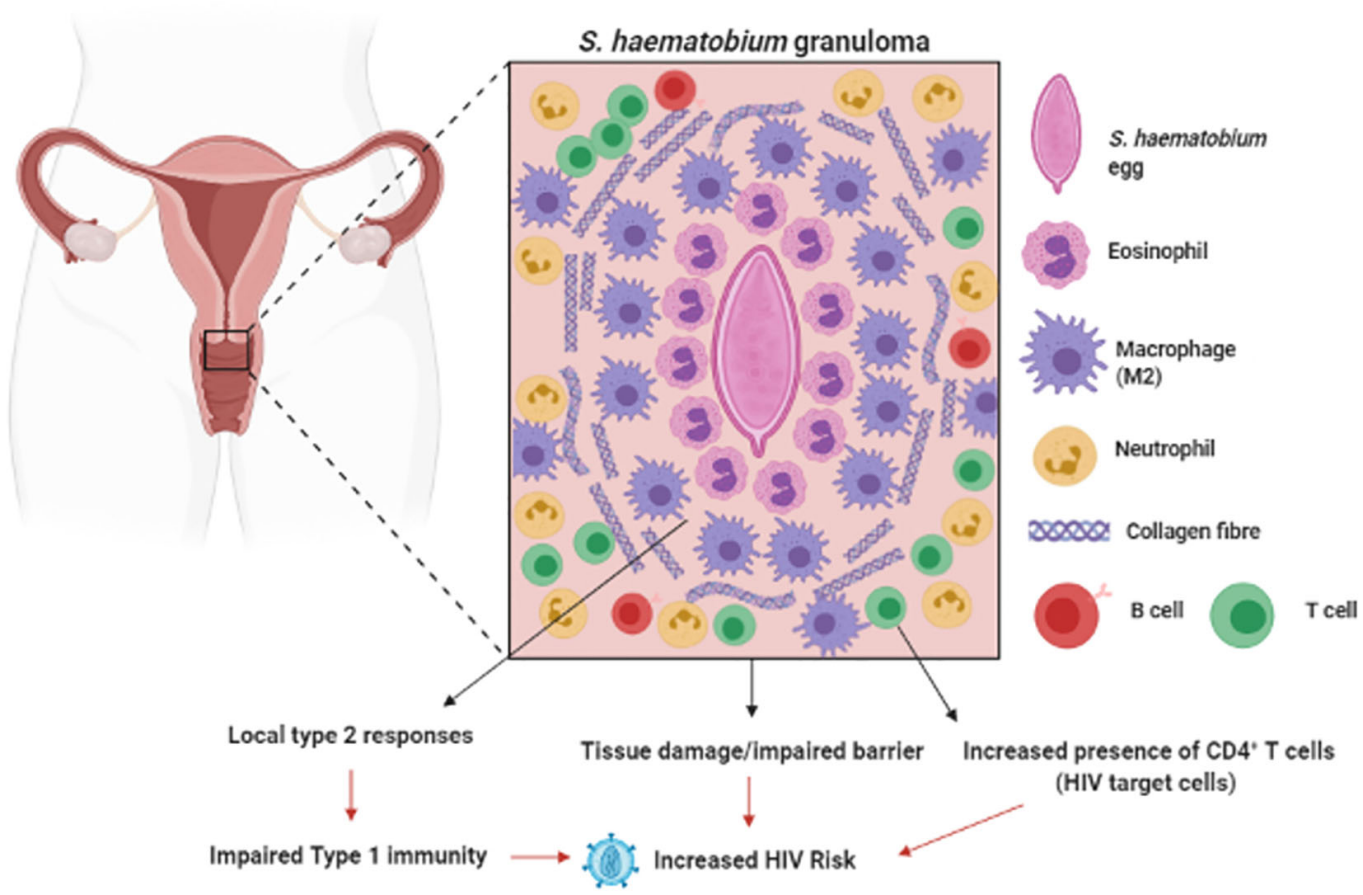
Genital Schistosomiasis: In S. *haematobium infected* women, eggs can become lodged in the cervix, resulting in inflammation around the schistosome eggs (granula) and bystanders tissue damage. Genital schistosomiasis is common and can impair vaginal immunity and increase Human Immunodeficiency Virus (HIV) risk (129–133). Created with BioRender.com.

In the mouse model of urinary schistosomiasis, injection of eggs into the urinary bladder results in formation of a granuloma around the eggs made up of neutrophils, eosinophils and macrophages, as well as the onset of fibrosis in the surrounding bladder tissue (127). Furthermore, in this model *S. haematobium* eggs induced a strong type-2 response characterized by eosinophilia and elevated IL-4, IL-13 and IL-5 in the tissue surrounding the eggs. A compromised FRT epithelium is associated with increased HIV risk (128). The bystander tissue damage resulting from *S. haematobium* egg-induced inflammation (129, 130), increased immune activation (131) and lesions in the FRT is reasonably hypothesized to increase host risk of HIV infection, by providing routes for viral entry and increased number of target cells at the site of infection (132) (**Figure 2**). Furthermore, the type 2 response induced during *S. haematobium* infection (127) may dampen type 1 responses required for protection against viral pathogens such as HIV. These hypotheses are supported by clinical findings, where women infected with *S. haematobium* may have up to a 3-fold increased risk of acquiring HIV (133–135).

Following treatment with the anti-helminthic drug, praziquantel, the immune response in treated individuals shifts from a type 2 and regulatory T cell immune response (131, 136, 137) to a pro-inflammatory state, with elevated levels of egg antigen-specific TNF-α, IL-6, IFN-γ, IL-12p70, IL-8 and Th17 cytokines (IL-17, IL-21, and IL-23) post-treatment (138). If this inflammatory state results in reduced susceptibility to HIV infection is yet to be explored.

### Filariasis

Filarial-driven immune modulation (i.e. induction of Th2, regulatory immune responses and suppression of inflammatory/Th1 responses) may increase susceptibility to viral and bacterial infections in the FRT, as Th1/inflammatory responses are important for the defense against these pathogens (139, 140). This is supported by identification of an association between infection with the filarial nematode *Wuchereria bancrofti* and increased risk of HIV infection (141). This increased HIV susceptibility may be associated with systemic increase in proportions of CD4^+^ T cells expressing HLA-DR and HLA-DR/CD38, as well as effector memory CD4^+^ T cells in lymphatic filariasis patients, i.e. an increase in HIV target cells in these patients (142). This supports *in vitro* findings demonstrating increased HIV infection of PBMC from lymphatic filariasis patients in comparison to uninfected individuals (12). Increased inflammation has also been reported in lymphatic filariasis patients (62, 143), with systemic IL-17 and IFN-γ elevated in response to PBMC stimulation with filarial antigen in these individuals. With chronic filarial infections, a type 2 immune signature, i.e. elevated IL-4 and IL-5, is detected in antigen-stimulated host PBMCs (143, 144). In contrast to schistosomiasis, regulatory T cells were reduced in lymphatic filariasis cases (62, 144) however type 1 responses (IFN-γ production) were suppressed in these patients (144). These studies suggest that chronic filarial infections could alter susceptibility to common FRT pathogens requiring type 1-mediated immune control. Surprisingly, genital manifestations of *W. bancrofti* infection have not been associated with any changes to fertility or pathology in the FRT (145).

### Soil-Transmitted Helminths

**Figure 3:**
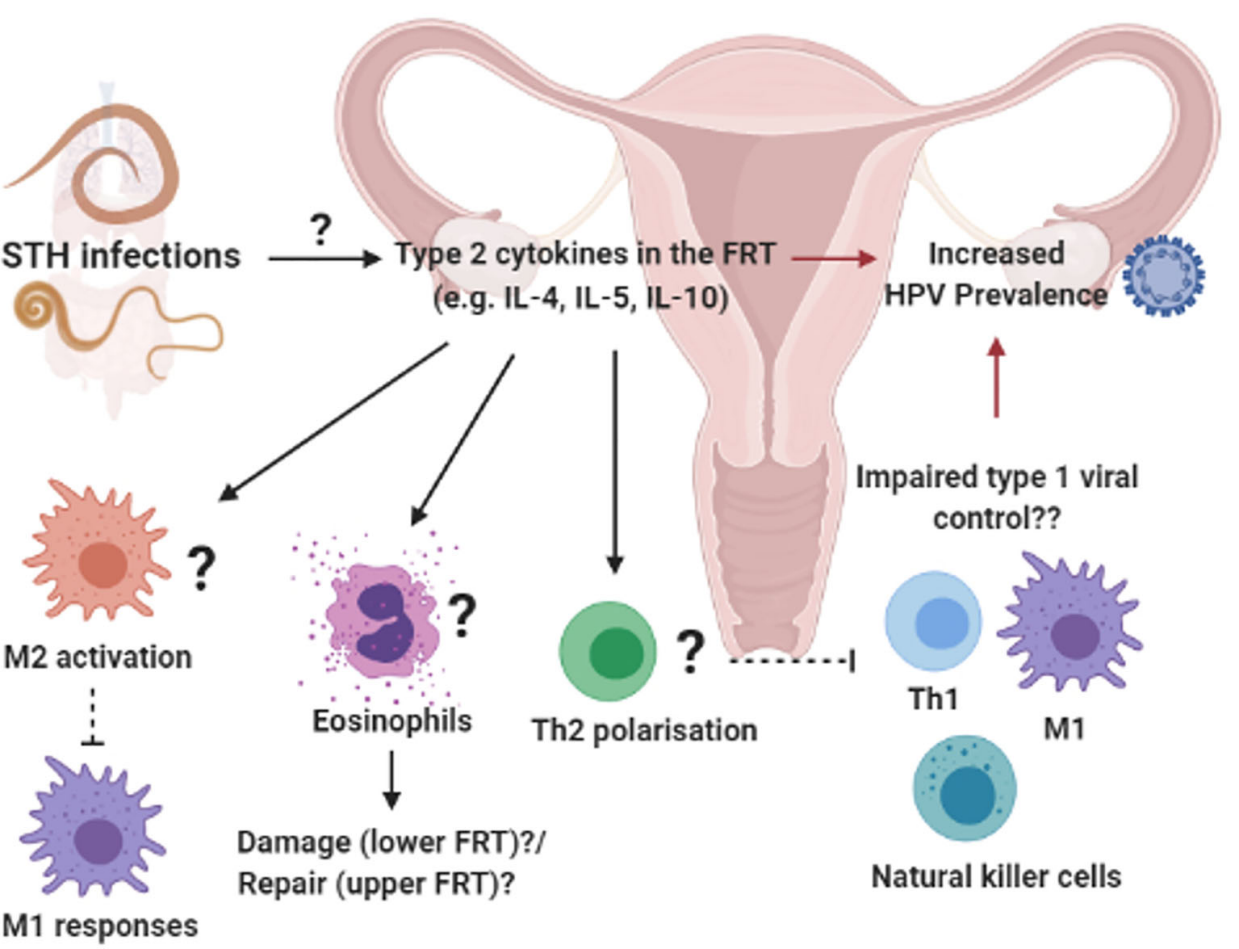
Systemic influences of soil-transmitted helminths on uncolonized female reproductive tract (FRT): Prevalent soil-transmitted helminth (STH) infections, which transit the lung and GIT, can systemically alter host immunity in biological compartments not directly colonized by the parasite. For example, STH exposure was associated with increased HPV risk and a helminth-associated type 2 cytokine profile in vagina fluid of women in a STH endemic region (87). We hypothesize that the induction of type 2 immunity in the FRT e.g. type 2 cytokines activating M2 macrophages, eosinophils an Th2 differentiation of CD4^+^ T cells, could impair protective type 1 immune responses and increased susceptibility to viral STIs (52, 147–151). Created with BioRender.com.

Unlike schistosomiasis that causes direct pathology to the FRT, evidence has emerged of the potential systemic effect of helminths at sites that are not colonized by these pathogens. In a STH endemic region of Peru, Gravitt et al. (87) reported an increased prevalence of HPV among older women (30–45 years old) infected with STHs, which included *T. trichiura, A. lumbricoides, Ancylostoma duodenale* and *Strongyloides stercoralis*. Importantly, the life cycle of these helminths does not involve any larval transit through, or egg deposition in the FRT. The type 2 cytokine IL-4 was detected in cervicovaginal lavages of these women and IL-4 levels correlated positively with other cytokines involved in anti-helminth immunity; IL-25, IL-21, IL-5, IL-10, IL-8, and IL-31 (87). The authors hypothesized that the increased HPV prevalence among older women in STH-endemic regions, is mediated by helminth-induced immune regulation which may impair viral control, supported by a *in vivo* studies which demonstrate IL-4-mediated impairment of anti-viral immunity (52, 78, 146) (**Figure 3**). This study therefore suggests a systemic skewing of the immune response towards a type 2 phenotype detectable in the FRT impairing host ability to control HPV *via* type 1-mediated mechanisms. In contrast, murine hookworm *N. brasiliensis* antigen has been shown to inhibit HPV-16 pseudovirion uptake by human cervical cell lines. Furthermore, murine hookworm antigen exposure and *in vivo* infection decreased expression of cell surface vimentin or total vimentin expression in the cell line or the FRT, respectively (152). Cell surface vimentin has previously been described as a restriction factor that mediates internalization of HPV pseudovirion particles (153). This suggests that helminth exposure may alter cervical epithelial susceptibility to HPV infection. Further, *N. brasiliensis* L3 somatic antigen decreased migration of cervical cancer cells in motility assays, suggesting a possible downmodulation of cancer cell metastasis by this helminth. Further studies are required to fully understand the complex consequences of helminth infection on HPV infection and pathogenesis.

### HPV, Cancer, and Type 2 Immunity

Persistent HPV strains evade protective host immune responses, which are the first steps to the development of high-grade cervical lesions and cancer (154–156). Interestingly, type 1/type 2 antagonism can be manipulated by oncogenic HPV, to suppress anti-viral responses, promote persistence and tumor development (157). For example, Lepique et al. (147) described an association between M2-like macrophages and the suppression of anti-tumor responses and tumor progression during HPV-related cancer (147). Here, they identified tumor-associated macrophages (TAMs) as a dominate population in tumors, with high baseline Arginase I and IL-10 expression, and low iNOS activity, when stimulated with LPS/IFN-γ. Additionally, Petrillo et al. (148) reported a correlation between increased ratio of M2:M1 macrophages and poor responses to treatment and survival (148). Regulatory cytokines IL-10, TGF-β and prostaglandin E2 (PGE_2_) produced by M2-like TAMs, promote the accumulation of regulatory T cells, which are associated with viral persistence and tumor development (158–160). Production of type 2 cytokines (e.g. IL-4, IL-13) by M2-like TAMs promotes Th2 polarization, reducing Th1 and CTL responses (149, 161–164). Moreover, Xie et al. (150) reported high levels of eosinophils in cervical cancer lesions and demonstrated that thymic stromal lymphopoietin (TSLP)-mediated eosinophil infiltration and activation promoted proliferation of cancer cells *in vitro* (150). Considering the significance of type1/type 2 imbalances during HPV persistence and related cancer progression, we hypothesize that helminth-induced type 2 immunity may impair anti-viral and anti-tumor immune responses, resulting in the promotion of tumor progression in the FRT (**Figure 3**).

### Type 2 Immunity in the FRT

The role of type 2 immunity in modulating immune responses in the FRT has been demonstrated by Oh et al. (165), where induction of the type 2-associated ‘alarmin’ IL-33 in the genital mucosa, increased susceptibility to the HSV-2 pathology *in vivo* (165). The mediator of this effect was vaginal dysbiosis, which promoted IL-33 and impaired recruitment of memory T cells and reduced IFN-γ production in the FRT. These mice also demonstrated marked eosinophil accumulation and elevated IL-5 in the FRT (165). Furthermore, administration of recombinant IL-33 or protease-mediated induction of IL-33 in the vagina resulted in heightened susceptibility to HSV-2 (165). Oh et al. (166) elaborated on this model of IL-33-mediated type 2 immune induction in the FRT, through administration of the serine protease papain. Here, papain-induced IL-33 in the vagina lead to the accumulation of vaginal eosinophils and production of canonical type 2 cytokines IL-4, IL-5, and IL-13 in genital lymph node T cells (166). Furthermore, elevated levels of type 2-associated IgE and IgG1 were detected in vaginal washes of papain-treated mice. Although elevated levels of IL-5 and eosinophils were detected in the FRT, papain induction of type 2 immunity in the FRT was not dependent on eosinophil recruitment, but rather on myeloid differentiation primary response gene 88 (MyD88) signaling and PDL2^+^CD301b^+^ dendritic cells under the control of interferon regulatory factor 4 (IRF4) (166).

Conversely, Vicetti Miguel et al. (151) demonstrated the protective role of type 2 immunity during *in vivo C. trachomatis* infection. *Chlamydia-*induced damage of the upper genital tract was prevented by IL-4 producing eosinophils, which promotes proliferation of endometrial stromal cells and tissue repair (151). Together, these studies demonstrate the significance of type 2 immunity in the FRT during STI infections and highlight potential differences in the role of type 2 responses at different sites in the FRT.

### Helminths and Fecundity

The dichotomy of type 1: type 2 immune responses has been studied during the stages of pregnancy and labor, with a type 2 bias contributing to immune tolerance and a successful pregnancy (167). This would suggest that type 2-inducing helminth infections may systemically influence pregnancy in infected mothers in a positive manner. Interestingly, *in vivo* studies have demonstrated that helminth infection can result in pregnancy loss and failure of implantation of fertilized eggs (168), as well as reduced fecundity in parasitized hosts (169). Using a *Schistosoma mansoni* mouse model, Straubinger et al. (68) demonstrated that infected female mice gave birth to pups with lower birth weights during the Th2 phase of the immune response, as opposed to uninfected mice (68). In humans, Kurtis et al. (170) reported an association between maternal schistosomiasis and increased levels of inflammatory cytokines in mothers’, placental and cord blood (170). As mother-to-child transmission of the schistosomes has not been reported in humans, the authors hypothesized the inflammatory response is likely due to helminth antigen movement across the placenta (170, 171). Furthermore, McDonald et al. (171) measured increased levels of pro-fibrotic proteins in the cord blood of neonates born to *S. japonicum-*infected mothers (171). Clinical trials by Ndibazza et al. (172) and Olveda et al. (173) reported that treatment of pregnant women in endemic regions with anti-Schistosome drug praziquantel, did not significantly alter birth outcomes (172–174).

For maternal STH infections, Blackwell et al. (175) reported an association between hookworm infection and delayed age of first pregnancy and lower odds of successive pregnancies after the initial pregnancy. The converse was observed with *Ascaris* infection, which positively associated with conception at a younger age and shortened intervals of subsequent pregnancies after the first, among women younger than 32 years of age living in helminth endemic regions (175). The authors hypothesized that the opposing observations in fecundity between hookworm and *Ascaris* infections, is associated with the differing immune responses to the parasites; *A. lumbricoides* is associated with a polarized Th2 response (37) whereas hookworm infections may induce a mixed Th1/Th2 response (176). Together these studies suggest that helminth infections can have profound effects on female reproductive health, experimental investigation is required to better understanding of these effects.

## Concluding Remarks

In this review, we have outlined the local and potential systemic effects of helminth infections on female reproductive health and susceptibility to STIs. Considering the great geographical overlap between STI and helminth prevalence, as well as the reduced access to health care and poor female health in helminth endemic regions, the study of helminth influences on the FRT should be a priority going forward, with focus on systemic effects of these parasites on uncolonized mucosal sites. Importantly, further comprehension on the systemic effects of GI helminths is needed, to direct health care strategies to mitigate the burden of helminth infections on the female reproductive health in those most at risk.

## Author Contribution

All authors contributed to the article and approved the submitted version.

## Conflict of Interest

The authors declare that the research was conducted in the absence of any commercial or financial relationships that could be construed as a potential conflict of interest.

## References

[1] de Silva NR Brooker S Hotez PJ Montresor A Engels D Savioli L Soil-transmitted helminth infections: updating the global picture. Trends Parasitol (2003) 19(12):547–51. 10.1016/j.pt.2003.10.002 14642761

[2] McSorley HJ O’Gorman MT Blair N Sutherland TE Filbey KJ Maizels RM Suppression of type 2 immunity and allergic airway inflammation by secreted products of the helminth Heligmosomoides polygyrus. Eur J Immunol (2012) 42(10):2667–82. 10.1002/eji.201142161 PMC4916998 22706967

[3] du Plessis N Kleynhans L Thiart L van Helden PD Brombacher F Horsnell WG Acute helminth infection enhances early macrophage mediated control of mycobacterial infection. Mucosal Immunol (2013) 6(5):931–41. 10.1038/mi.2012.131 23250274

[4] Bobat S Darby M Mrdjen D Cook C Logan E Auret J Natural and vaccine-mediated immunity to Salmonella Typhimurium is impaired by the helminth Nippostrongylus brasiliensis. PLoS Negl Trop Dis (2014) 8(12):e3341. 10.1371/journal.pntd.0003341 25474738 PMC4256288

[5] Jõgi NO Svanes C Siiak SP Logan E Holloway JW Igland J Zoonotic helminth exposure and risk of allergic diseases: A study of two generations in Norway. Clin Exp Allergy (2018) 48(1):66–77. 10.1111/cea.13055 29117468

[6] O’Shea MK Fletcher TE Muller J Tanner R Matsumiya M Bailey JW Human Hookworm Infection Enhances Mycobacterial Growth Inhibition and Associates With Reduced Risk of Tuberculosis Infection. Front Immunol (2018) 9:2893. 10.3389/fimmu.2018.02893 30619265 PMC6302045

[7] Rolot M Dougall AM Chetty A Javaux J Chen T Xiao X Helminth-induced IL-4 expands bystander memory CD8(+) T cells for early control of viral infection. Nat Commun (2018) 9(1):4516. 10.1038/s41467-018-06978-5 30375396 PMC6207712

[8] Organizations WH Sexually transmitted infections: implementing the Global STI Strategy: Evidence-to-action brief. WHO reference number: WHO/RHR/17.18. Geneva, Switzerland: World Health Organization (2017).

[9] Ivan E Crowther NJ Rucogoza AT Osuwat LO Munyazesa E Mutimura E Malaria and helminthic co-infection among HIV-positive pregnant women: prevalence and effects of antiretroviral therapy. Acta Trop (2012) 124(3):179–84. 10.1016/j.actatropica.2012.08.004 22940013

[10] Mkhize-Kwitshana ZL Taylor M Jooste P Mabaso MLH Walzl G The influence of different helminth infection phenotypes on immune responses against HIV in co-infected adults in South Africa. BMC Infect Dis (2011) 11(1):273. 10.1186/1471-2334-11-273 21999928 PMC3213674

[11] Abossie A Petros B Deworming and the immune status of HIV positive pre-antiretroviral therapy individuals in Arba Minch, Chencha and Gidole hospitals, Southern Ethiopia. BMC Res Notes (2015) 8:483. 10.1186/s13104-015-1461-9 26415705 PMC4585997

[12] Gopinath R Ostrowski M Justement SJ Fauci AS Nutman TB Filarial infections increase susceptibility to human immunodeficiency virus infection in peripheral blood mononuclear cells *in vitro* . J Infect Dis (2000) 182(6):1804–8. 10.1086/317623 11069260

[13] Wolday D Mayaan S Mariam ZG Berhe N Seboxa T Britton S Treatment of intestinal worms is associated with decreased HIV plasma viral load. J Acquired Immune Defic Syndromes (1999) (2002) 31(1):56–62. 10.1097/00126334-200209010-00008 12352151

[14] Brown M Mawa PA Joseph S Bukusuba J Watera C Whitworth JA Treatment of Schistosoma mansoni infection increases helminth-specific type 2 cytokine responses and HIV-1 loads in coinfected Ugandan adults. J Infect Dis (2005) 191(10):1648–57. 10.1086/429668 15838791

[15] Camberis M Le Gros G Urban J Jr. Animal model of Nippostrongylus brasiliensis and Heligmosomoides polygyrus. Curr Protoc Immunol (2003) Chapter 19:Unit 19.2. 10.1002/0471142735.im1912s55 18432905

[16] Lawrence RA Devaney E Lymphatic filariasis: parallels between the immunology of infection in humans and mice. Parasit Immunol (2001) 23(7):353–61. 10.1046/j.1365-3024.2001.00396.x 11472555

[17] Cliffe LJ Grencis RK The Trichuris muris system: a paradigm of resistance and susceptibility to intestinal nematode infection. Adv Parasitol (2004) 57:255–307. 10.1016/S0065-308X(04)57004-5 15504540

[18] Lewis R Behnke JM Stafford P Holland CV The development of a mouse model to explore resistance and susceptibility to early Ascaris suum infection. Parasitology (2006) 132(Pt 2):289–300. 10.1017/S0031182005008978 16209722

[19] Shea-Donohue T Sullivan C Finkelman FD Madden KB Morris SC Goldhill J The role of IL-4 in Heligmosomoides polygyrus-induced alterations in murine intestinal epithelial cell function. J Immunol (Baltimore Md: 1950) (2001) 167(4):2234–9. 10.4049/jimmunol.167.4.2234 11490010

[20] Madden KB Whitman L Sullivan C Gause WC Urban JF Jr. Katona IM Role of STAT6 and mast cells in IL-4- and IL-13-induced alterations in murine intestinal epithelial cell function. J Immunol (Baltimore Md: 1950) (2002) 169(8):4417–22. 10.4049/jimmunol.169.8.4417 12370375

[21] Horsnell WG Cutler AJ Hoving JC Mearns H Myburgh E Arendse B Delayed goblet cell hyperplasia, acetylcholine receptor expression, and worm expulsion in SMC-specific IL-4Ralpha-deficient mice. PLoS Pathog (2007) 3(1):e1. 10.1371/journal.ppat.0030001 17222057 PMC1769405

[22] Mearns H Horsnell WG Hoving JC Dewals B Cutler AJ Kirstein F Interleukin-4-promoted T helper 2 responses enhance Nippostrongylus brasiliensis-induced pulmonary pathology. Infect Immun (2008) 76(12):5535–42. 10.1128/IAI.00210-08 PMC2583554 18809669

[23] Horsnell WGC Vira A Kirstein F Mearns H Hoving JC Cutler AJ IL-4Rα-responsive smooth muscle cells contribute to initiation of TH2 immunity and pulmonary pathology in Nippostrongylus brasiliensis infections. Mucosal Immunol (2010) 4:83. 10.1038/mi.2010.46 20737001

[24] Schmidt S Hoving JC Horsnell WGC Mearns H Cutler AJ Brombacher TM Nippostrongylus-Induced Intestinal Hypercontractility Requires IL-4 Receptor Alpha-Responsiveness by T Cells in Mice. PLoS One (2012) 7(12):e52211. 10.1371/journal.pone.0052211 23284939 PMC3527412

[25] Thawer SG Horsnell WG Darby M Hoving JC Dewals B Cutler AJ Lung-resident CD4+ T cells are sufficient for IL-4Rα-dependent recall immunity to Nippostrongylus brasiliensis infection. Mucosal Immunol (2013) 7:239. 10.1038/mi.2013.40 23778354

[26] Finkelman FD Shea-Donohue T Morris SC Gildea L Strait R Madden KB Interleukin-4- and interleukin-13-mediated host protection against intestinal nematode parasites. Immunol Rev (2004) 201:139–55. 10.1111/j.0105-2896.2004.00192.x 15361238

[27] Neill DR Wong SH Bellosi A Flynn RJ Daly M Langford TK Nuocytes represent a new innate effector leukocyte that mediates type-2 immunity. Nature (2010) 464(7293):1367–70. 10.1038/nature08900 PMC2862165 20200518

[28] Klose CS Artis D Innate lymphoid cells as regulators of immunity, inflammation and tissue homeostasis. Nat Immunol (2016) 17(7):765–74. 10.1038/ni.3489 27328006

[29] Nussbaum JC Van Dyken SJ von Moltke J Cheng LE Mohapatra A Molofsky AB Type 2 innate lymphoid cells control eosinophil homeostasis. Nature (2013) 502(7470):245–8. 10.1038/nature12526 PMC3795960 24037376

[30] Patnode ML Bando JK Krummel MF Locksley RM Rosen SD Leukotriene B4 amplifies eosinophil accumulation in response to nematodes. J Exp Med (2014) 211(7):1281–8. 10.1084/jem.20132336 PMC4076593 24889202

[31] Knott ML Matthaei KI Foster PS Dent LA The roles of eotaxin and the STAT6 signalling pathway in eosinophil recruitment and host resistance to the nematodes Nippostrongylus brasiliensis and Heligmosomoides bakeri. Mol Immunol (2009) 46(13):2714–22. 10.1016/j.molimm.2009.05.016 19535141

[32] Ganley-Leal LM Mwinzi PN Cetre-Sossah CB Andove J Hightower AW Karanja DM Correlation between eosinophils and protection against reinfection with Schistosoma mansoni and the effect of human immunodeficiency virus type 1 coinfection in humans. Infect Immun (2006) 74(4):2169–76. 10.1128/IAI.74.4.2169-2176.2006 PMC1418888 16552047

[33] Zhao A McDermott J Urban JF Gause W Madden KB Yeung KA Dependence of IL-4, IL-13, and Nematode-Induced Alterations in Murine Small Intestinal Smooth Muscle Contractility on Stat6 and Enteric Nerves. J Immunol (2003) 171(2):948–54. 10.4049/jimmunol.171.2.948 12847266

[34] Zhao A Urban JF Jr. Anthony RM Sun R Stiltz J van Rooijen N Th2 cytokine-induced alterations in intestinal smooth muscle function depend on alternatively activated macrophages. Gastroenterology (2008) 135(1):217–25.e1. 10.1053/j.gastro.2008.03.077 18471439 PMC2954589

[35] Turner JD Faulkner H Kamgno J Cormont F Van Snick J Else KJ Th2 cytokines are associated with reduced worm burdens in a human intestinal helminth infection. J Infect Dis (2003) 188(11):1768–75. 10.1086/379370 14639550

[36] Jackson JA Turner JD Rentoul L Faulkner H Behnke JM Hoyle M Cytokine response profiles predict species-specific infection patterns in human GI nematodes. Int J Parasitol (2004) 34(11):1237–44. 10.1016/j.ijpara.2004.07.009 15491586

[37] Geiger SM Massara CL Bethony J Soboslay PT Carvalho OS Corrêa-Oliveira R Cellular responses and cytokine profiles in Ascaris lumbricoides and Trichuris trichiura infected patients. Parasit Immunol (2002) 24(11-12):499–509. 10.1046/j.1365-3024.2002.00600.x 12694600

[38] Jackson JA Turner JD Rentoul L Faulkner H Behnke JM Hoyle M T helper cell type 2 responsiveness predicts future susceptibility to gastrointestinal nematodes in humans. J Infect Dis (2004) 190(10):1804–11. 10.1086/425014 15499537

[39] Quinnell RJ Pritchard DI Raiko A Brown AP Shaw MA Immune responses in human necatoriasis: association between interleukin-5 responses and resistance to reinfection. J Infect Dis (2004) 190(3):430–8. 10.1086/422256 15243914

[40] Gaze S McSorley HJ Daveson J Jones D Bethony JM Oliveira LM Characterising the mucosal and systemic immune responses to experimental human hookworm infection. PLoS Pathog (2012) 8(2):e1002520–e. 10.1371/journal.ppat.1002520 PMC3276555 22346753

[41] Grainger JR Smith KA Hewitson JP McSorley HJ Harcus Y Filbey KJ Helminth secretions induce *de novo* T cell Foxp3 expression and regulatory function through the TGF-beta pathway. J Exp Med (2010) 207(11):2331–41. 10.1084/jem.20101074 PMC2964568 20876311

[42] Johnston CJC Smyth DJ Kodali RB White MPJ Harcus Y Filbey KJ A structurally distinct TGF-β mimic from an intestinal helminth parasite potently induces regulatory T cells. Nat Commun (2017) 8(1):1741. 10.1038/s41467-017-01886-6 29170498 PMC5701006

[43] Maizels RM Smits HH McSorley HJ Modulation of Host Immunity by Helminths: The Expanding Repertoire of Parasite Effector Molecules. Immunity (2018) 49(5):801–18. 10.1016/j.immuni.2018.10.016 PMC6269126 30462997

[44] McSorley HJ Harcus YM Murray J Taylor MD Maizels RM Expansion of Foxp3+ regulatory T cells in mice infected with the filarial parasite Brugia malayi. J Immunol (Baltimore Md: 1950) (2008) 181(9):6456–66. 10.4049/jimmunol.181.9.6456 18941236

[45] Watanabe K Mwinzi PN Black CL Muok EM Karanja DM Secor WE T regulatory cell levels decrease in people infected with Schistosoma mansoni on effective treatment. Am J Trop Med Hyg (2007) 77(4):676–82. 10.4269/ajtmh.2007.77.676 PMC2602861 17978070

[46] Doetze A Satoguina J Burchard G Rau T Loliger C Fleischer B Antigen-specific cellular hyporesponsiveness in a chronic human helminth infection is mediated by T(h)3/T(r)1-type cytokines IL-10 and transforming growth factor-beta but not by a T(h)1 to T(h)2 shift. Int Immunol (2000) 12(5):623–30. 10.1093/intimm/12.5.623 10784608

[47] Satoguina J Mempel M Larbi J Badusche M Löliger C Adjei O Antigen-specific T regulatory-1 cells are associated with immunosuppression in a chronic helminth infection (onchocerciasis). Microbes Infect (2002) 4(13):1291–300. 10.1016/S1286-4579(02)00014-X 12443893

[48] Ricci ND Fiúza JA Bueno LL Cançado GG Gazzinelli-Guimarães PH Martins VG Induction of CD4(+)CD25(+)FOXP3(+) regulatory T cells during human hookworm infection modulates antigen-mediated lymphocyte proliferation. PLoS Negl Trop Dis (2011) 5(11):e1383. 10.1371/journal.pntd.0001383 22087344 PMC3210756

[49] Mosmann TR Cherwinski H Bond MW Giedlin MA Coffman RL Two types of murine helper T cell clone. I. Definition according to profiles of lymphokine activities and secreted proteins. J Immunol (Baltimore Md: 1950) (1986) 136(7):2348–57. 2419430

[50] Mosmann TR Coffman RL TH1 and TH2 cells: different patterns of lymphokine secretion lead to different functional properties. Annu Rev Immunol (1989) 7:145–73. 10.1146/annurev.iy.07.040189.001045 2523712

[51] Fernandez-Botran R Sanders VM Mosmann TR Vitetta ES Lymphokine-mediated regulation of the proliferative response of clones of T helper 1 and T helper 2 cells. J Exp Med (1988) 168(2):543–58. 10.1084/jem.168.2.543 PMC2189014 2970518

[52] Reese TA Wakeman BS Choi HS Hufford MM Huang SC Zhang X Helminth infection reactivates latent -herpesvirus *via* cytokine competition at a viral promoter. Science (2014) 345(6196):573–7. 10.1126/science.1254517 PMC4531374 24968940

[53] Briken V Mosser DM Editorial: switching on arginase in M2 macrophages. J Leukoc Biol (2011) 90(5):839–41. 10.1189/jlb.0411203 PMC3206469 22045920

[54] Taylor MD Harris A Nair MG Maizels RM Allen JE F4/80+ alternatively activated macrophages control CD4+ T cell hyporesponsiveness at sites peripheral to filarial infection. J Immunol (Baltimore Md: 1950 (2006) 176(11):6918–27. 10.4049/jimmunol.176.11.6918 16709852

[55] Smith KA Filbey KJ Reynolds LA Hewitson JP Harcus Y Boon L Low-level regulatory T-cell activity is essential for functional type-2 effector immunity to expel gastrointestinal helminths. Mucosal Immunol (2016) 9(2):428–43. 10.1038/mi.2015.73 PMC4677460 26286232

[56] Finney CA Taylor MD Wilson MS Maizels RM Expansion and activation of CD4(+)CD25(+) regulatory T cells in Heligmosomoides polygyrus infection. Eur J Immunol (2007) 37(7):1874–86. 10.1002/eji.200636751 PMC2699425 17563918

[57] Bancroft AJ Levy CW Jowitt TA Hayes KS Thompson S McKenzie EA The major secreted protein of the whipworm parasite tethers to matrix and inhibits interleukin-13 function. Nat Commun (2019) 10(1):2344–. 10.1038/s41467-019-09996-z PMC6538607 31138806

[58] Ritter M Osei-Mensah J Debrah LB Kwarteng A Mubarik Y Debrah AY Wuchereria bancrofti-infected individuals harbor distinct IL-10-producing regulatory B and T cell subsets which are affected by anti-filarial treatment. PLoS Negl Trop Dis (2019) 13(5):e0007436. 10.1371/journal.pntd.0007436 31120872 PMC6550419

[59] Sartono E Kruize YC Kurniawan A van der Meide PH Partono F Maizels RM Elevated cellular immune responses and interferon-gamma release after long-term diethylcarbamazine treatment of patients with human lymphatic filariasis. J Infect Dis (1995) 171(6):1683–7. 10.1093/infdis/171.6.1683 7769319

[60] Mahanty S Mollis SN Ravichandran M Abrams JS Kumaraswami V Jayaraman K High levels of spontaneous and parasite antigen-driven interleukin-10 production are associated with antigen-specific hyporesponsiveness in human lymphatic filariasis. J Infect Dis (1996) 173(3):769–73. 10.1093/infdis/173.3.769 8627051

[61] King CL Mahanty S Kumaraswami V Abrams JS Regunathan J Jayaraman K Cytokine control of parasite-specific anergy in human lymphatic filariasis. Preferential induction of a regulatory T helper type 2 lymphocyte subset. J Clin Invest (1993) 92(4):1667–73. 10.1172/JCI116752 PMC288325 8408619

[62] Babu S Bhat SQ Kumar NP Jayantasri S Rukmani S Kumaran P Human type 1 and 17 responses in latent tuberculosis are modulated by coincident filarial infection through cytotoxic T lymphocyte antigen-4 and programmed death-1. J Infect Dis (2009) 200(2):288–98. 10.1086/599797 PMC2997351 19505258

[63] Katawa G Layland LE Debrah AY von Horn C Batsa L Kwarteng A Hyperreactive onchocerciasis is characterized by a combination of Th17-Th2 immune responses and reduced regulatory T cells. PLoS Negl Trop Dis (2015) 9(1):e3414. 10.1371/journal.pntd.0003414 25569210 PMC4288720

[64] Li Y Guan X Liu W Chen H-L Truscott J Beyatli S Helminth-Induced Production of TGF-β and Suppression of Graft-versus-Host Disease Is Dependent on IL-4 Production by Host Cells. J Immunol (2018) 201(10):2910–22. 10.4049/jimmunol.1700638 PMC6219912 30291167

[65] Ferreira IB Pickering DA Troy S Croese J Loukas A Navarro S Suppression of inflammation and tissue damage by a hookworm recombinant protein in experimental colitis. Clin Trans Immunol (2017) 6(10):e157. 10.1038/cti.2017.42 PMC5671989 29114386

[66] Navarro S Pickering DA Ferreira IB Jones L Ryan S Troy S Hookworm recombinant protein promotes regulatory T cell responses that suppress experimental asthma. Sci Trans Med (2016) 8(362):362ra143–362ra143. 10.1126/scitranslmed.aaf8807 27797959

[67] Layland LE Straubinger K Ritter M Loffredo-Verde E Garn H Sparwasser T Schistosoma mansoni-Mediated Suppression of Allergic Airway Inflammation Requires Patency and Foxp3+ Treg Cells. PLoS Negl Trop Dis (2013) 7(8):e2379. 10.1371/journal.pntd.0002379 23967364 PMC3744427

[68] Straubinger K Paul S Prazeres da Costa O Ritter M Buch T Busch DH Maternal immune response to helminth infection during pregnancy determines offspring susceptibility to allergic airway inflammation. J Allergy Clin Immunol (2014) 134(6):1271–9.e10. 10.1016/j.jaci.2014.05.034 25042744

[69] Osbourn M Soares DC Vacca F Cohen ES Scott IC Gregory WF HpARI Protein Secreted by a Helminth Parasite Suppresses Interleukin-33. Immunity (2017) 47(4):739–51.e5. 10.1016/j.immuni.2017.09.015 29045903 PMC5655542

[70] Zaiss Mario M Rapin A Lebon L Dubey Lalit K Mosconi I Sarter K The Intestinal Microbiota Contributes to the Ability of Helminths to Modulate Allergic Inflammation. Immunity (2015) 43(5):998–1010. 10.1016/j.immuni.2015.09.012 26522986 PMC4658337

[71] Pinelli E Brandes S Dormans J Gremmer E van Loveren H Infection with the roundworm Toxocara canis leads to exacerbation of experimental allergic airway inflammation. Clin Exp Allergy (2008) 38(4):649–58. 10.1111/j.1365-2222.2007.02908.x 18167123

[72] Darby MG Chetty A Mrjden D Rolot M Smith K Mackowiak C Pre-conception maternal helminth infection transfers *via* nursing long-lasting cellular immunity against helminths to offspring. Sci Adv (2019) 5(5):eaav3058. 10.1126/sciadv.aav3058 31236458 PMC6587632

[73] Sabin EA Araujo MI Carvalho EM Pearce EJ Impairment of tetanus toxoid-specific Th1-like immune responses in humans infected with Schistosoma mansoni. J Infect Dis (1996) 173(1):269–72. 10.1093/infdis/173.1.269 8537675

[74] Cooper PJ Chico M Sandoval C Espinel I Guevara A Levine MM Human infection with Ascaris lumbricoides is associated with suppression of the interleukin-2 response to recombinant cholera toxin B subunit following vaccination with the live oral cholera vaccine CVD 103-HgR. Infect Immun (2001) 69(3):1574–80. 10.1128/IAI.69.3.1574-1580.2001 PMC98058 11179329

[75] Elias D Wolday D Akuffo H Petros B Bronner U Britton S Effect of deworming on human T cell responses to mycobacterial antigens in helminth-exposed individuals before and after bacille Calmette-Guerin (BCG) vaccination. Clin Exp Immunol (2001) 123(2):219–25. 10.1046/j.1365-2249.2001.01446.x PMC1905995 11207651

[76] Nookala S Srinivasan S Kaliraj P Narayanan RB Nutman TB Impairment of tetanus-specific cellular and humoral responses following tetanus vaccination in human lymphatic filariasis. Infect Immun (2004) 72(5):2598–604. 10.1128/IAI.72.5.2598-2604.2004 PMC387878 15102768

[77] Apiwattanakul N Thomas PG Iverson AR McCullers JA Chronic helminth infections impair pneumococcal vaccine responses. Vaccine (2014) 32(42):5405–10. 10.1016/j.vaccine.2014.07.107 25131738

[78] Osborne LC Monticelli LA Nice TJ Sutherland TE Siracusa MC Hepworth MR Virus-helminth coinfection reveals a microbiota-independent mechanism of immunomodulation. Science (2014) 345(6196):578–82. 10.1126/science.1256942 PMC4548887 25082704

[79] McFarlane AJ McSorley HJ Davidson DJ Fitch PM Errington C Mackenzie KJ Enteric helminth-induced type I interferon signaling protects against pulmonary virus infection through interaction with the microbiota. J Allergy Clin Immunol (2017) 140(4):1068–78.e6. 10.1016/j.jaci.2017.01.016 28196762 PMC6485385

[80] Furze RC Hussell T Selkirk ME Amelioration of Influenza-Induced Pathology in Mice by Coinfection with Trichinella spiralis. Infect Immun (2006) 74(3):1924–32. 10.1128/IAI.74.3.1924-1932.2006 PMC1418664 16495568

[81] Metenou S Babu S Nutman TB Impact of filarial infections on coincident intracellular pathogens: Mycobacterium tuberculosis and Plasmodium falciparum. Curr Opin HIV AIDS (2012) 7(3):231–8. 10.1097/COH.0b013e3283522c3d PMC3431797 22418448

[82] Rajamanickam A Munisankar S Bhootra Y Dolla CK Nutman TB Babu S Coexistent Helminth Infection-Mediated Modulation of Chemokine Responses in Latent Tuberculosis. J Immunol (Baltimore Md: 1950) (2019) 202(5):1494–500. 10.4049/jimmunol.1801190 PMC6382527 30651341

[83] Chard AN Baker KK Tsai K Levy K Sistrunk JR Chang HH Associations between soil-transmitted helminthiasis and viral, bacterial, and protozoal enteroinfections: a cross-sectional study in rural Laos. Parasit Vectors (2019) 12(1):216. 10.1186/s13071-019-3471-2 31064387 PMC6505259

[84] Yasuda K Adachi T Koida A Nakanishi K Nematode-Infected Mice Acquire Resistance to Subsequent Infection With Unrelated Nematode by Inducing Highly Responsive Group 2 Innate Lymphoid Cells in the Lung. Front Immunol (2018) 9:2132. 10.3389/fimmu.2018.02132 30283458 PMC6157322

[85] Filbey KJ Camberis M Chandler J Turner R Kettle AJ Eichenberger RM Intestinal helminth infection promotes IL-5- and CD4+ T cell-dependent immunity in the lung against migrating parasites. Mucosal Immunol (2019) 12(2):352–62. 10.1038/s41385-018-0102-8 30401814

[86] Price AE Liang H-E Sullivan BM Reinhardt RL Eisley CJ Erle DJ Systemically dispersed innate IL-13–expressing cells in type 2 immunity. Proc Natl Acad Sci (2010) 107(25):11489–94. 10.1073/pnas.1003988107 PMC2895098 20534524

[87] Gravitt PE Marks M Kosek M Huang C Cabrera L Olortegui MP Soil-Transmitted Helminth Infections Are Associated With an Increase in Human Papillomavirus Prevalence and a T-Helper Type 2 Cytokine Signature in Cervical Fluids. J Infect Dis (2016) 213(5):723–30. 10.1093/infdis/jiv498 PMC4747620 26486638

[88] Borgdorff H Gautam R Armstrong SD Xia D Ndayisaba GF van Teijlingen NH Cervicovaginal microbiome dysbiosis is associated with proteome changes related to alterations of the cervicovaginal mucosal barrier. Mucosal Immunol (2016) 9(3):621–33. 10.1038/mi.2015.86 26349657

[89] Wira CR Fahey JV Rodriguez-Garcia M Shen Z Patel MV Regulation of mucosal immunity in the female reproductive tract: the role of sex hormones in immune protection against sexually transmitted pathogens. Am J Reprod Immunol (2014) 72(2):236–58. 10.1111/aji.12252 PMC4351777 24734774

[90] Valore EV Park CH Igreti SL Ganz T Antimicrobial components of vaginal fluid. Am J Obstet Gynecol (2002) 187(3):561–8. 10.1067/mob.2002.125280 12237628

[91] Kaushic C HIV-1 Infection in the Female Reproductive Tract: Role of Interactions between HIV-1 and Genital Epithelial Cells. Am J Reprod Immunol (2011) 65(3):253–60. 10.1111/j.1600-0897.2010.00965.x 21223427

[92] Chan T Barra NG Lee AJ Ashkar AA Innate and adaptive immunity against herpes simplex virus type 2 in the genital mucosa. J Reprod Immunol (2011) 88(2):210–8. 10.1016/j.jri.2011.01.001 21334750

[93] Iwasaki A Mucosal dendritic cells. Annu Rev Immunol (2007) 25:381–418. 10.1146/annurev.immunol.25.022106.141634 17378762

[94] Zhao X Deak E Soderberg K Linehan M Spezzano D Zhu J Vaginal submucosal dendritic cells, but not Langerhans cells, induce protective Th1 responses to herpes simplex virus-2. J Exp Med (2003) 197(2):153–62. 10.1084/jem.20021109 PMC2193810 12538655

[95] Perez-Zsolt D Cantero-Pérez J Erkizia I Benet S Pino M Serra-Peinado C Dendritic Cells From the Cervical Mucosa Capture and Transfer HIV-1 *via* Siglec-1. Front Immunol (2019) 10(825). 10.3389/fimmu.2019.00825 PMC6503733 31114569

[96] Spellberg B Edwards JE Jr. Type 1/Type 2 immunity in infectious diseases. Clin Infect Dis (2001) 32(1):76–102. 10.1086/317537 11118387

[97] Shin HD Winkler C Stephens JC Bream J Young H Goedert JJ Genetic restriction of HIV-1 pathogenesis to AIDS by promoter alleles of IL10. Proc Natl Acad Sci U S A (2000) 97(26):14467–72. 10.1073/pnas.97.26.14467 PMC18942 11121048

[98] McGuirk P Mills KH Pathogen-specific regulatory T cells provoke a shift in the Th1/Th2 paradigm in immunity to infectious diseases. Trends Immunol (2002) 23(9):450–5. 10.1016/S1471-4906(02)02288-3 12200067

[99] Bettahi I Zhang X Afifi RE BenMohamed L Protective immunity to genital herpes simplex virus type 1 and type 2 provided by self-adjuvanting lipopeptides that drive dendritic cell maturation and elicit a polarized Th1 immune response. Viral Immunol (2006) 19(2):220–36. 10.1089/vim.2006.19.220 16817765

[100] Scott M Stites DP Moscicki AB Th1 cytokine patterns in cervical human papillomavirus infection. Clin Diagn Lab Immunol (1999) 6(5):751–5. 10.1128/CDLI.6.5.751-755.1999 PMC95767 10473530

[101] Maloy KJ Burkhart C Junt TM Odermatt B Oxenius A Piali L CD4(+) T cell subsets during virus infection. Protective capacity depends on effector cytokine secretion and on migratory capability. J Exp Med (2000) 191(12):2159–70. 10.1084/jem.191.12.2159 PMC2193195 10859340

[102] Goldszmid Romina S Caspar P Rivollier A White S Dzutsev A Hieny S NK Cell-Derived Interferon-γ Orchestrates Cellular Dynamics and the Differentiation of Monocytes into Dendritic Cells at the Site of Infection. Immunity (2012) 36(6):1047–59. 10.1016/j.immuni.2012.03.026 PMC3412151 22749354

[103] Lee AJ Chen B Chew MV Barra NG Shenouda MM Nham T Inflammatory monocytes require type I interferon receptor signaling to activate NK cells *via* IL-18 during a mucosal viral infection. J Exp Med (2017) 214(4):1153–67. 10.1084/jem.20160880 PMC5379971 28264883

[104] Butz EA Bevan MJ Massive expansion of antigen-specific CD8+ T cells during an acute virus infection. Immunity (1998) 8(2):167–75. 10.1016/S1074-7613(00)80469-0 PMC2776648 9491998

[105] Dobbs ME Strasser JE Chu CF Chalk C Milligan GN Clearance of herpes simplex virus type 2 by CD8+ T cells requires gamma interferon and either perforin- or Fas-mediated cytolytic mechanisms. J Virol (2005) 79(23):14546–54. 10.1128/JVI.79.23.14546-14554.2005 PMC1287581 16282454

[106] Koelle DM Posavad CM Barnum GR Johnson ML Frank JM Corey L Clearance of HSV-2 from recurrent genital lesions correlates with infiltration of HSV-specific cytotoxic T lymphocytes. J Clin Invest (1998) 101(7):1500–8. 10.1172/JCI1758 PMC508728 9525993

[107] Deng K Pertea M Rongvaux A Wang L Durand CM Ghiaur G Broad CTL response is required to clear latent HIV-1 due to dominance of escape mutations. Nature (2015) 517(7534):381–5. 10.1038/nature14053 PMC4406054 25561180

[108] Murthy AK Li W Chaganty BKR Kamalakaran S Guentzel MN Seshu J Tumor necrosis factor alpha production from CD8+ T cells mediates oviduct pathological sequelae following primary genital Chlamydia muridarum infection. Infect Immun (2011) 79(7):2928–35. 10.1128/IAI.05022-11 PMC3191981 21536799

[109] Jordan SJ Gupta K Ogendi BMO Bakshi RK Kapil R Press CG The Predominant CD4(+) Th1 Cytokine Elicited to Chlamydia trachomatis Infection in Women Is Tumor Necrosis Factor Alpha and Not Interferon Gamma. Clin Vaccine Immunol (2017) 24(4):e00010–17. 10.1128/CVI.00010-17 PMC5382828 28100498

[110] Borrow P Lewicki H Hahn BH Shaw GM Oldstone MB Virus-specific CD8+ cytotoxic T-lymphocyte activity associated with control of viremia in primary human immunodeficiency virus type 1 infection. J Virol (1994) 68(9):6103–10. 10.1128/JVI.68.9.6103-6110.1994 PMC237022 8057491

[111] Masson L Passmore J-AS Liebenberg LJ Werner L Baxter C Arnold KB Genital inflammation and the risk of HIV acquisition in women. Clin Infect Dis (2015) 61(2):260–9. 10.1093/cid/civ298 PMC4565995 25900168

[112] Ganatra SR Bucsan AN Alvarez X Kumar S Chatterjee A Quezada M Anti-retroviral therapy does not reduce tuberculosis reactivation in a tuberculosis-HIV co-infection model. J Clin Invest (2020) 130(10):5171–9. 10.1172/JCI136502 PMC7524506 32544085

[113] Borges ÁH Combination antiretroviral therapy and cancer risk. Curr Opin HIV AIDS (2017) 12(1):12–9. 10.1097/COH.0000000000000334 PMC5244841 27755153

[114] Feinen B Jerse AE Gaffen SL Russell MW Critical role of Th17 responses in a murine model of Neisseria gonorrhoeae genital infection. Mucosal Immunol (2010) 3(3):312–21. 10.1038/mi.2009.139 PMC2857675 20107432

[115] Vasilevsky S Greub G Nardelli-Haefliger D Baud D Genital Chlamydia trachomatis: understanding the roles of innate and adaptive immunity in vaccine research. Clin Microbiol Rev (2014) 27(2):346–70. 10.1128/CMR.00105-13 PMC3993100 24696438

[116] WHO Guidelines Approved by the Guidelines Review Committee WHO Guidelines for the Treatment of Chlamydia trachomatis. Geneva: World Health Organization Copyright © World Health Organization 2016 (2016).

[117] Poston TB Darville T Chlamydia trachomatis: Protective Adaptive Responses and Prospects for a Vaccine. Curr Topics Microbiol Immunol (2018) 412:217–37. 10.1007/82_2016_6 27033698

[118] Vicetti Miguel RD Quispe Calla NE Pavelko SD Cherpes TL Intravaginal Chlamydia trachomatis Challenge Infection Elicits TH1 and TH17 Immune Responses in Mice That Promote Pathogen Clearance and Genital Tract Damage. PLoS One (2016) 11(9):e0162445–e. 10.1371/journal.pone.0162445 PMC5015975 27606424

[119] Kanhere A Hertweck A Bhatia U Gökmen MR Perucha E Jackson I T-bet and GATA3 orchestrate Th1 and Th2 differentiation through lineage-specific targeting of distal regulatory elements. Nat Commun (2012) 3(1):1268. 10.1038/ncomms2260 23232398 PMC3535338

[120] Zhou L Lopes JE Chong MM Ivanov II Min R Victora GD TGF-beta-induced Foxp3 inhibits T(H)17 cell differentiation by antagonizing RORgammat function. Nature (2008) 453(7192):236–40. 10.1038/nature06878 PMC2597437 18368049

[121] Odegaard JI Hsieh MH Immune responses to Schistosoma haematobium infection. Parasit Immunol (2014) 36(9):428–38. 10.1111/pim.12084 25201406

[122] Hegertun IEA Sulheim Gundersen KM Kleppa E Zulu SG Gundersen SG Taylor M S. haematobium as a Common Cause of Genital Morbidity in Girls: A Cross-sectional Study of Children in South Africa. PLoS Negl Trop Dis (2013) 7(3):e2104. 10.1371/journal.pntd.0002104 23556009 PMC3605138

[123] Norseth HM Ndhlovu PD Kleppa E Randrianasolo BS Jourdan PM Roald B The Colposcopic Atlas of Schistosomiasis in the Lower Female Genital Tract Based on Studies in Malawi, Zimbabwe, Madagascar and South Africa. PLoS Negl Trop Dis (2014) 8(11):e3229. 10.1371/journal.pntd.0003229 25412334 PMC4238986

[124] Ismail HAHA Hong S-T Babiker ATEB Hassan RMAE Sulaiman MAZ Jeong H-G Prevalence, risk factors, and clinical manifestations of schistosomiasis among school children in the White Nile River basin, Sudan. Parasit Vectors (2014) 7:478. 10.1186/s13071-014-0478-6 25312470 PMC4200116

[125] Randrianasolo BS Jourdan PM Ravoniarimbinina P Ramarokoto CE Rakotomanana F Ravaoalimalala VE Gynecological manifestations, histopathological findings, and schistosoma-specific polymerase chain reaction results among women with Schistosoma haematobium infection: a cross-sectional study in Madagascar. J Infect Dis (2015) 212(2):275–84. 10.1093/infdis/jiv035 PMC4482143 25725656

[126] Ishida K Hsieh MH Understanding Urogenital Schistosomiasis-Related Bladder Cancer: An Update. Front Med (2018) 5:223. 10.3389/fmed.2018.00223 PMC6104441 30159314

[127] Fu CL Odegaard JI Herbert DR Hsieh MH A novel mouse model of Schistosoma haematobium egg-induced immunopathology. PLoS Pathog (2012) 8(3):e1002605. 10.1371/journal.ppat.1002605 22479181 PMC3315496

[128] Looker KJ Elmes JAR Gottlieb SL Schiffer JT Vickerman P Turner KME Effect of HSV-2 infection on subsequent HIV acquisition: an updated systematic review and meta-analysis. Lancet Infect Dis (2017) 17(12):1303–16. 10.1016/S1473-3099(17)30405-X PMC5700807 28843576

[129] Wright ED Chiphangwi J Hutt MS Schistosomiasis of the female genital tract. A histopathological study of 176 cases from Malawi. Trans R Soc Trop Med Hyg (1982) 76(6):822–9. 10.1016/0035-9203(82)90118-3 7164149

[130] Helling-Giese G Sjaastad A Poggensee G Kjetland EF Richter J Chitsulo L Female genital schistosomiasis (FGS): relationship between gynecological and histopathological findings. Acta Trop (1996) 62(4):257–67. 10.1016/S0001-706X(96)00027-7 9028410

[131] Nausch N Midzi N Mduluza T Maizels RM Mutapi F Regulatory and activated T cells in human Schistosoma haematobium infections. PLoS One (2011) 6(2):e16860. 10.1371/journal.pone.0016860 21347311 PMC3037381

[132] Klatt NR Chomont N Douek DC Deeks SG Immune activation and HIV persistence: implications for curative approaches to HIV infection. Immunol Rev (2013) 254(1):326–42. 10.1111/imr.12065 PMC3694608 23772629

[133] Kjetland EF Ndhlovu PD Gomo E Mduluza T Midzi N Gwanzura L Association between genital schistosomiasis and HIV in rural Zimbabwean women. AIDS (Lond Engl) (2006) 20(4):593–600. 10.1097/01.aids.0000210614.45212.0a 16470124

[134] Ndhlovu PD Mduluza T Kjetland EF Midzi N Nyanga L Gundersen SG Prevalence of urinary schistosomiasis and HIV in females living in a rural community of Zimbabwe: does age matter? Trans R Soc Trop Med Hyg (2007) 101(5):433–8. 10.1016/j.trstmh.2006.08.008 17064746

[135] Downs JA Mguta C Kaatano GM Mitchell KB Bang H Simplice H Urogenital schistosomiasis in women of reproductive age in Tanzania’s Lake Victoria region. Am J Trop Med Hyg (2011) 84(3):364–9. 10.4269/ajtmh.2011.10-0585 PMC3042809 21363971

[136] He YX Chen L Ramaswamy K Schistosoma mansoni, S. haematobium, and S. japonicum: early events associated with penetration and migration of schistosomula through human skin. Exp Parasitol (2002) 102(2):99–108. 10.1016/S0014-4894(03)00024-9 12706745

[137] Kullberg MC Pearce EJ Hieny SE Sher A Berzofsky JA Infection with Schistosoma mansoni alters Th1/Th2 cytokine responses to a non-parasite antigen. J Immunol (Baltimore Md: 1950) (1992) 148(10):3264–70. 1533656

[138] Bourke CD Nausch N Rujeni N Appleby LJ Mitchell KM Midzi N Integrated analysis of innate, Th1, Th2, Th17, and regulatory cytokines identifies changes in immune polarisation following treatment of human schistosomiasis. J Infect Dis (2013) 208(1):159–69. 10.1093/infdis/jis524 PMC3666130 23045617

[139] Allen JE Adjei O Bain O Hoerauf A Hoffmann WH Makepeace BL Of mice, cattle, and humans: the immunology and treatment of river blindness. PLoS Negl Trop Dis (2008) 2(4):e217–e. 10.1371/journal.pntd.0000217 PMC2323618 18446236

[140] Hoerauf A Brattig N Resistance and susceptibility in human onchocerciasis–beyond Th1 vs. Th2. Trends Parasitol (2002) 18(1):25–31. 10.1016/S1471-4922(01)02173-0 11850011

[141] Kroidl I Saathoff E Maganga L Makunde WH Hoerauf A Geldmacher C Effect of Wuchereria bancrofti infection on HIV incidence in southwest Tanzania: a prospective cohort study. Lancet (Lond Engl) (2016) 388(10054):1912–20. 10.1016/S0140-6736(16)31252-1 27495354

[142] Kroidl I Chachage M Mnkai J Nsojo A Berninghoff M Verweij JJ Wuchereria bancrofti infection is linked to systemic activation of CD4 and CD8 T cells. PLoS Negl Trop Dis (2019) 13(8):e0007623. 10.1371/journal.pntd.0007623 31425508 PMC6736309

[143] Arndts K Deininger S Specht S Klarmann U Mand S Adjobimey T Elevated adaptive immune responses are associated with latent infections of Wuchereria bancrofti. PLoS Negl Trop Dis (2012) 6(4):e1611. 10.1371/journal.pntd.0001611 22509424 PMC3317915

[144] Babu S Kumaraswami V Nutman TB Transcriptional control of impaired Th1 responses in patent lymphatic filariasis by T-box expressed in T cells and suppressor of cytokine signaling genes. Infect Immun (2005) 73(6):3394–401. 10.1128/IAI.73.6.3394-3401.2005 PMC1111868 15908366

[145] Bernhard P Makunde RW Magnussen P Lemnge MM Genital manifestations and reproductive health in female residents of a Wuchereria bancrofti-endemic area in Tanzania. Trans R Soc Trop Med Hyg (2000) 94(4):409–12. 10.1016/S0035-9203(00)90123-8 11127246

[146] Veldhoen M Heeney JL A helminth-mediated viral awakening. Trends Immunol (2014) 35(10):452–3. 10.1016/j.it.2014.08.004 25174993

[147] Lepique AP Daghastanli KR Cuccovia IM Villa LL HPV16 tumor associated macrophages suppress antitumor T cell responses. Clin Cancer Res (2009) 15(13):4391–400. 10.1158/1078-0432.CCR-09-0489 19549768

[148] Petrillo M Zannoni GF Martinelli E Pedone Anchora L Ferrandina G Tropeano G Polarisation of Tumor-Associated Macrophages toward M2 Phenotype Correlates with Poor Response to Chemoradiation and Reduced Survival in Patients with Locally Advanced Cervical Cancer. PLoS One (2015) 10(9):e0136654. 10.1371/journal.pone.0136654 26335330 PMC4559430

[149] Feng Q Wei H Morihara J Stern J Yu M Kiviat N Th2 type inflammation promotes the gradual progression of HPV-infected cervical cells to cervical carcinoma. Gynecol Oncol (2012) 127(2):412–9. 10.1016/j.ygyno.2012.07.098 PMC3472044 22828962

[150] Xie F Liu L-B Shang W-Q Chang K-K Meng Y-H Mei J The infiltration and functional regulation of eosinophils induced by TSLP promote the proliferation of cervical cancer cell. Cancer Lett (2015) 364(2):106–17. 10.1016/j.canlet.2015.04.029 25979231

[151] Vicetti Miguel RD Quispe Calla NE Dixon D Foster RA Gambotto A Pavelko SD IL-4–secreting eosinophils promote endometrial stromal cell proliferation and prevent Chlamydia-induced upper genital tract damage. Proc Natl Acad Sci (2017) 114(33):E6892–E901. 10.1073/pnas.1621253114 PMC5565408 28765368

[152] Jacobs BA Chetty A Horsnell WGC Schafer G Prince S Smith KA Hookworm exposure decreases human papillomavirus uptake and cervical cancer cell migration through systemic regulation of epithelial-mesenchymal transition marker expression. Sci Rep (2018) 8(1):11547. 10.1038/s41598-018-30058-9 30069018 PMC6070561

[153] Schäfer G Graham LM Lang DM Blumenthal MJ Bergant Marušič M Katz AA Vimentin Modulates Infectious Internalization of Human Papillomavirus 16 Pseudovirions. J Virol (2017) 91(16):e00307–17. 10.1128/JVI.00307-17 PMC5533935 28566373

[154] Guess JC McCance DJ Decreased Migration of Langerhans Precursor-Like Cells in Response to Human Keratinocytes Expressing Human Papillomavirus Type 16 E6/E7 Is Related to Reduced Macrophage Inflammatory Protein-3α Production. J Virol (2005) 79(23):14852–62. 10.1128/JVI.79.23.14852-14862.2005 PMC1287574 16282485

[155] Pahne-Zeppenfeld J Schröer N Walch-Rückheim B Oldak M Gorter A Hegde S Cervical cancer cell-derived interleukin-6 impairs CCR7-dependent migration of MMP-9-expressing dendritic cells. Int J Cancer (2014) 134(9):2061–73. 10.1002/ijc.28549 24136650

[156] Garcia-Iglesias T del Toro-Arreola A Albarran-Somoza B del Toro-Arreola S Sanchez-Hernandez PE Ramirez-Dueñas MG NKp30, NKp46 and NKG2D expression and reduced cytotoxic activity on NK cells in cervical cancer and precursor lesions. Low BMC Cancer (2009) 9(1):186. 10.1186/1471-2407-9-186 19531227 PMC2704222

[157] Zhou C Tuong ZK Frazer IH Papillomavirus Immune Evasion Strategies Target the Infected Cell and the Local Immune System. Front Oncol (2019) 9(682). 10.3389/fonc.2019.00682 PMC6688195 31428574

[158] Loddenkemper C Hoffmann C Stanke J Nagorsen D Baron U Olek S Regulatory (FOXP3+) T cells as target for immune therapy of cervical intraepithelial neoplasia and cervical cancer. Cancer Sci (2009) 100(6):1112–7. 10.1111/j.1349-7006.2009.01153.x PMC11159425 19514119

[159] Kim KH Greenfield WW Cannon MJ Coleman HN Spencer HJ Nakagawa M CD4+ T-cell response against human papillomavirus type 16 E6 protein is associated with a favorable clinical trend. Cancer Immunol Immunother: CII (2012) 61(1):63–70. 10.1007/s00262-011-1092-5 21842207 PMC3374341

[160] Molling JW de Gruijl TD Glim J Moreno M Rozendaal L Meijer CJ CD4(+)CD25hi regulatory T-cell frequency correlates with persistence of human papillomavirus type 16 and T helper cell responses in patients with cervical intraepithelial neoplasia. Int J Cancer (2007) 121(8):1749–55. 10.1002/ijc.22894 17582606

[161] al-Saleh W Giannini SL Jacobs N Moutschen M Doyen J Boniver J Correlation of T-helper secretory differentiation and types of antigen-presenting cells in squamous intraepithelial lesions of the uterine cervix. J Pathol (1998) 184(3):283–90. 10.1002/(SICI)1096-9896(199803)184:3<283::AID-PATH25>3.0.CO;2-K 9614381

[162] Clerici M Merola M Ferrario E Trabattoni D Villa ML Stefanon B Cytokine production patterns in cervical intraepithelial neoplasia: association with human papillomavirus infection. J Natl Cancer Inst (1997) 89(3):245–50. 10.1093/jnci/89.3.245 9017005

[163] Bais AG Beckmann I Lindemans J Ewing PC Meijer CJLM Snijders PJF A shift to a peripheral Th2-type cytokine pattern during the carcinogenesis of cervical cancer becomes manifest in CIN III lesions. J Clin Pathol (2005) 58(10):1096–100. 10.1136/jcp.2004.025072 PMC1770745 16189158

[164] Lee B-N Follen M Tortolero-Luna G Eriksen N Helfgott A Hammill H Synthesis of IFN-γ by CD8+ T Cells Is Preserved in HIV-Infected Women with HPV-Related Cervical Squamous Intraepithelial Lesions. Gynecol Oncol (1999) 75(3):379–86. 10.1006/gyno.1999.5587 10600293

[165] Oh JE Kim B-C Chang D-H Kwon M Lee SY Kang D Dysbiosis-induced IL-33 contributes to impaired antiviral immunity in the genital mucosa. Proc Natl Acad Sci (2016) 113(6):E762–E71. 10.1073/pnas.1518589113 PMC4760794 26811463

[166] Oh JE Oh DS Jung HE Lee HK A mechanism for the induction of type 2 immune responses by a protease allergen in the genital tract. Proc Natl Acad Sci (2017) 114(7):E1188–E95. 10.1073/pnas.1612997114 PMC5320955 28137851

[167] Sykes L MacIntyre DA Yap XJ Teoh TG Bennett PR The Th1:th2 dichotomy of pregnancy and preterm labour. Mediators Inflamm (2012) 2012:967629. 10.1155/2012/967629 22719180 PMC3376783

[168] Krishnan L Guilbert LJ Wegmann TG Belosevic M Mosmann TR T helper 1 response against Leishmania major in pregnant C57BL/6 mice increases implantation failure and fetal resorptions. Correlation with increased IFN-gamma and TNF and reduced IL-10 production by placental cells. J Immunol (Baltimore Md: 1950) (1996) 156(2):653–62. 8543817

[169] Hurd H Host fecundity reduction: a strategy for damage limitation? Trends Parasitol (2001) 17(8):363–8. 10.1016/S1471-4922(01)01927-4 11685895

[170] Kurtis JD Higashi A Wu HW Gundogan F McDonald EA Sharma S Maternal Schistosomiasis japonica is associated with maternal, placental, and fetal inflammation. Infect Immun (2011) 79(3):1254–61. 10.1128/IAI.01072-10 PMC3067505 21149589

[171] McDonald EA Cheng L Jarilla B Sagliba MJ Gonzal A Amoylen AJ Maternal infection with Schistosoma japonicum induces a profibrotic response in neonates. Infect Immun (2014) 82(1):350–5. 10.1128/IAI.01060-13 PMC3911825 24166958

[172] Ndibazza J Muhangi L Akishule D Kiggundu M Ameke C Oweka J Effects of deworming during pregnancy on maternal and perinatal outcomes in Entebbe, Uganda: a randomized controlled trial. Clin Infect Dis (2010) 50(4):531–40. 10.1086/649924 PMC2857962 20067426

[173] Olveda RM Acosta LP Tallo V Baltazar PI Lesiguez JL Estanislao GG Efficacy and safety of praziquantel for the treatment of human schistosomiasis during pregnancy: a phase 2, randomised, double-blind, placebo-controlled trial. Lancet Infect Dis (2016) 16(2):199–208. 10.1016/S1473-3099(15)00345-X 26511959 PMC4752899

[174] Friedman JF Olveda RM Mirochnick MH Bustinduy AL Elliott AM Praziquantel for the treatment of schistosomiasis during human pregnancy. Bull World Health Organ (2018) 96(1):59–65. 10.2471/BLT.17.198879 29403101 PMC5791873

[175] Blackwell AD Tamayo MA Beheim B Trumble BC Stieglitz J Hooper PL Helminth infection, fecundity, and age of first pregnancy in women. Science (2015) 350(6263):970–2. 10.1126/science.aac7902 PMC5953513 26586763

[176] Geiger SM Alexander ND Fujiwara RT Brooker S Cundill B Diemert DJ Necator americanus and helminth co-infections: further down-modulation of hookworm-specific type 1 immune responses. PLoS Negl Trop Dis (2011) 5(9):e1280. 10.1371/journal.pntd.0001280 21909439 PMC3167770

